# Geochemical-Compositional-Functional Changes in Arctic Soil Microbiomes Post Land Submergence Revealed by Metagenomics

**DOI:** 10.1264/jsme2.ME18091

**Published:** 2019-06-07

**Authors:** Nengfei Wang, Yudong Guo, Gaoyang Li, Yan Xia, Mingyang Ma, Jiaye Zang, Yue Ma, Xiaofei Yin, Wenbing Han, Jinjiang Lv, Huansheng Cao

**Affiliations:** 1 Key Lab of Marine Bioactive Substances, First Institute of Oceanography, State Oceanic Administration Qingdao 266061 China; 2 Department of Bioengineering, College of Marine Sciences and Biological Engineering, Qingdao University of Science & Technology Qingdao 266042 China; 3 College of Computer Science and Technology, Jilin University Changchun, Jilin 100012 China; 4 Jilin University First Hospital Changchun, Jilin 100012 China; 5 College of Chemistry and Chemical Engineering, Qingdao University Qingdao 266071 China; 6 Center for Fundamental and Applied Microbiomics, Biodesign Institute, Arizona State University Tempe, AZ 85287 USA

**Keywords:** 16S rRNA gene, the Arctic, soil microbiomes, metagenome, meltwater

## Abstract

Lakes of meltwater in the Artic have become one of the transforming landscape changes under global warming. We herein compared microbial communities between sediments and bank soils at an arctic lake post land submergence using geochemistry, 16S rRNA amplicons, and metagenomes. The results obtained showed that each sample had approximately 2,609 OTUs on average and shared 1,716 OTUs based on the 16S rRNA gene V3–V4 region. Dominant phyla in sediments and soils included *Proteobacteria*, *Acidobacteria*, *Actinobacteria*, *Gemmatimonadetes*, and *Nitrospirae*; sediments contained a unique phylum, *Euryarchaeota*, with the phylum *Thaumarchaeota* being primarily present in bank soils. Among the top 35 genera across all sites, 17 were more abundant in sediments, while the remaining 18 were more abundant in bank soils; seven out of the top ten genera across all sites were only from sediments. A redundancy analysis separated sediment samples from soil samples based on the components of nitrite and ammonium. Metagenome results supported the role of nitrite because most of the genes for denitrification and methane metabolic genes were more abundant in sediments than in soils, while the abundance of phosphorus-utilizing genes was similar and, thus, was not a significant explanatory factor. We identified several modules from the global networks of OTUs that were closely related to some geochemical factors, such as pH and nitrite. Collectively, the present results showing consistent changes in geochemistry, microbiome compositions, and functional genes suggest an ecological mechanism across molecular and community levels that structures microbiomes post land submergence.

Climate change has profoundly affected landscapes globally, and its effects have been particularly apparent in the Arctic, at which a direct anthropogenic impact is minimal (19, 27). Among the most significant landscape changes is the formation of lakes or ponds from ice meltwater (36), which not only exposes soils that used to be covered by ice, but also submerges some land, creating a broad spectrum of local heterogeneous ecosystems (19, 50). Some lakes in the permafrost tundra have started to shrink and even disappear from Arctic high regions (41), and thaw lakes are forming or expanding in low arctic or subarctic regions (33). Lakes, particularly newly formed/forming ponds, represent another route by which soil biogeochemistry may be altered by global climate change (1).

The Arctic soil microbiota, the diversity of which is presumably as high as that of tropical forests (11), is a sensitive indicator that has exhibited rapid changes under global warming (44). Only two studies have been conducted on thaw lakes in the Canadian High Arctic (13, 14). Few studies have compared submerged soil communities by thaw water with unsubmerged soils, the latter of which have been extensively examined among the Arctic soils (13, 14, 20, 24, 43). Moreover, limited information is currently available on the geochemical factors shaping microbiome compositions and functions.

Mechanistically, microbial communities are structured by interactions between geochemical conditions and microbial capabilities. For example, a laboratory simulation of permafrost thaw showed that microbial metagenomes quickly reached convergence (being more similar to one another than when they were frozen), and the levels of several genes involved in the cycling of nitrogen and carbon changed during thaw (29). Other examples of metagenomic changes have also been reported in arctic peat soils (11, 20, 24). Furthermore, soil microbiomes appear to be associated with an array of environmental factors. Significant geochemical factors affecting microbiome assembly include organic carbon, moisture, pH, phosphorus (17, 34, 45), redox potential and oxidative stress (25, 28), nanoparticles (21), and temperature (5).

In the present study, we aimed to investigate microbiome structures and functions in lake sediments under melted water and soils in bank slopes. A thaw lake surrounded by exposed bank slopes was selected (Fig. 1). Samples were collected along the soil continuum from the bank top to sediments in order to examine the diversity and function of microbial communities as well as the geochemical parameters associated with them, as assessed by 16S rRNA gene, network, multi-variate correlation, and metagenomic analyses.

## Materials and Methods

### Study sites and sample collection

**Fig. 1:**
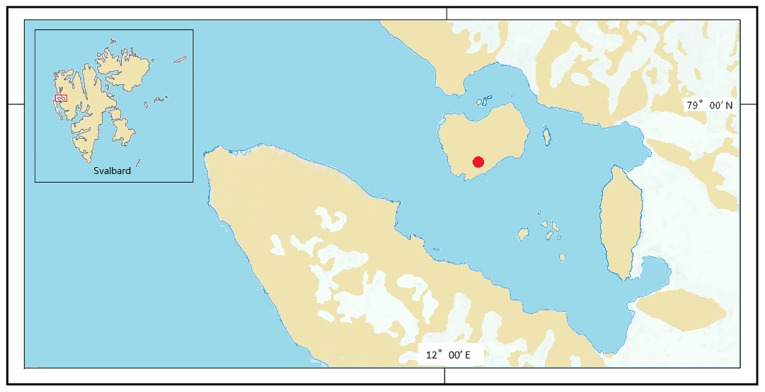
Study sites in the Arctic. The red dots represent the locations of the study sites; their position in the Svalbard archipelago is indicated in the inset.

The thaw lake studied was on the London Island of Konsfjorden on the west coast of Spitsbergen, the Svalbard archipelago (Fig. 1 and S1). This shallow pond (~2 m deep) is frozen to the bottom in winter and is supplied with water from melted pond ice and snow on the shore. In summer, the lake thaws and ice melts, and sediment samples were collected during this time. Sediments were sampled from a location submerged underwater all year round. Sediment post-submergence means sediment under melted lake water. On a transect (30 m) extending from pond sediments to the top of the bank, one sediment site (depth of 0.5 m; Sed16, 78°47′5057′47.30″N and 12°04′08.19″E) and three soil sites (Hil16, 78°57′46.82″N and 12°04′02.85″E; Up16, 78°57′46.94″N and 12°04′06.28″E; and Dow16, 78°57′46.86″N and 12°04′05.99″E) were selected. Surface soils and sediments (0–5 cm) were collected in triplicate (approximately 1 m from each other) and placed into TWIRL’EM sterile sampling bags (Labplas, Sainte-Julie, QC, Canada) in July 2016. Samples were stored at −80°C at China’s Arctic Yellow River Station before being transported on ice by air to the home laboratory, at which they were stored at −80°C until DNA extraction.

### Geochemical properties of soils and lake sediments

The moisture content (MC), pH, and total organic carbon (TOC), total organic nitrogen (TON), NO_3_^−^-N, NO_2_^−^-N, NH_4_^+^-N, SiO_4_^−^-Si, and PO_4_^3−^-P concentrations of the samples were measured as described previously (3). Soil pH was measured by adding 10 mL of distilled water to 4 g of soil for pH measurements using a pH meter (PHS-3C; Shanghai REX Instrument Factory, Shanghai, China). Soil samples were freeze-dried, ground into powder, treated with 10% HCl, and then dried in order to analyze TOC and TON using an element analyzer (EA3000; EuroVector, PAVIA, Italy). The soil samples used to assess nutrients were also freeze-dried and ground, and water was then added at a ratio of 1:10 (g mL^−1^). After shaking once every 4 h for 48 h, a nutrient auto-analyzer (QuAAtro, SEAL, Germany) was used to assess other physical and chemical properties relative to a standard deviation <5%.

### DNA extraction and 16S rRNA gene amplicon sequencing and analyses

DNA was extracted from an aliquot of 0.25 g soil/sediment of each sample using a PowerSoil DNA Isolation Kit (MO BIO Laboratories, San Diego, CA, USA) according to the manufacturer’s instructions. DNA extracted from samples was checked for integrity and contamination on 1% agarose gels and also for purity based on OD_260_/OD_280_ and OD_260_/OD_230_ using a NanoPhotometer spectrophotometer (IMPLEN, CA, USA). DNA concentrations were measured using the Qubit dsDNA Assay Kit in Qubit 2.0 Fluorometer (Life Technologies, CA, USA).

The V3 and V4 hypervariable regions of the bacterial 16S ribosomal RNA gene were amplified by PCR (98°C for 1 min, followed by 30 cycles at 98°C for 10 s, 50°C for 30 s, and 72°C for 30 s and a final extension at 72°C for 5 min) using the primers 341F 5′-barcode- CCTAYGGGRBGCASCAG-3′ (29) and 806R 5′-barcode-GGAC TACHVGGGTWTCTAAT-3′ (30), with barcodes being 8-base sequences unique to each sample. Sequencing libraries were prepared using the NEB Next Ultra DNA Library Prep Kit for Illumina (New England Biolabs, Ipswich, MA, USA) (47). Library quality was assessed on the Agilent Bioanalyzer 2100 system. Libraries were sequenced on an Illumina MiSeq platform and 300-bp paired-end reads were generated. Raw reads were deposited into the NCBI Sequence Read Archive database (accession number: SRP127650).

Paired-end reads from the original DNA fragments were merged with FLASH using default parameters (31) and assigned to each sample according to unique barcodes. These reads were then filtered using QIIME 1.8.0 (6) with the criteria specified by Wang *et al*. (47). Operational taxonomic units (OTUs) were clustered with a 97% similarity cut-off using UPARSE (10); chimeric sequences and singleton OTUs were identified and removed using UCHIME (9). The taxonomy of 16S rRNA gene sequences was assigned with a RDP Classifier (49) based on the Silva rRNA gene database release 128 at an 80% confidence threshold.

The diversity of each sample in terms of Chao1, Good’s coverage estimator, and Shannon’s inde alpha x (H’) were computed in QIIME 1.8.0 (6). The Kruskal-Wallis test was performed for the geochemical properties and diversity parameters of samples to assess the level of significance in R v3.3.1 (https://cran.r-project.org/). The bacterial communities of the 12 samples were investigated by a hierarchical clustering analysis in R. Beta diversity metrics, such as Unifrac distance, UPGMA clustering, and a Principal Coordinate Analysis (PCoA), were computed to examine dissimilarities between samples. The relevance of geochemical parameters in explaining the distribution patterns of bacterial communities was analyzed by a redundancy analysis (RDA) and sample-unique species were identified using Adonis in R package vegan (8). The taxonomic hierarchy of OTUs was shown in GraPhlAn (22).

### Identification of modules associated with geochemical parameters

To identify relationships between geochemical parameters and microbiomes, a network analysis was used to detect modules (sub-networks of OTUs) that correlate with geochemical factors. As described by Guidi *et al*. (16), the correlations of OTUs with seven out of nine geochemical parameters were assessed using the sparse partial least square (sPLS) (39) as implemented in the R package mixOmics (35). TON and PO_4_^3−^-P were excluded because their concentrations were below detection levels in 7 samples. A global network of OTUs based on relative abundance from all samples was constructed and modules were identified based on relationship (*i.e*., the correlation of a gene to a module eigengene, which is a 1-dimensional vector that summarizes or is representative of the relative abundance of the nodes in a module) the degree and their correlation with geochemical factors was assessed using the R package WGCNA (12). Modules were then exported, analyzed, and visualized in Cytoscape 3.6 (38).

### Metagenomic sequencing and analyses

A total of 1.0 μg DNA per sample was used for library construction with a NEBNext Ultra DNA Library Prep Kit for Illumina (New England Biolabs, Ipswich, MA, USA) following the manufacturer’s recommendations and corresponding index sequences. Briefly, DNA samples were fragmented by sonication to 350 bp, and fragments were end-polished, A-tailed, and ligated with the full-length adaptor for PCR amplification. PCR products were purified (AMPure XP system; Beckman Coulter, Brea, CA, USA), and libraries were analyzed for size distribution by an Agilent 2100 Bioanalyzer and quantified using real-time PCR. The clustering of index-coded samples was performed on a cBot Cluster Generation System according to the manufacturer’s instructions. After cluster generation, library preparations were sequenced on an Illumina HiSeq 2000 and paired-end reads were generated and deposited to NCBI SRA (accession number: SRP346710284).

In the metagenome analysis, reads of low quality (containing 40 or more bases with scores <20) or with more than 10 unknown bases (Ns) were removed using SOAPaligner (37). Clean reads from each sample were assembled using SOAPdenovo2 using default parameters (15). The reads not assembled into scaftigs (continuous sequences within scaffolds) in each sample were pooled together to form a mixed sample, which was reassembled. All scaftigs shorter than 500 bp were discarded.

Open reading frames in the scaftigs of each sample and of the mixed sample were predicted using MetaGeneMark (26). Redundant genes in each sample were removed using CD-HIT with 95% identity and 90% coverage (51). The resulting genes were further filtered with the number of reads assembled, and those assembled with less than two reads were discarded to generate a gene catalog (unigenes). Unigenes were then filtered using DIAMOND (23) against the reference genes in the NCBI-nr database at a cut-off e-value of 1E-10. In the functional analysis, DIAMOND (26) was used to map unigenes against reference genes in KEGG (4).

## Results

### Geochemical properties of soil and sediment samples

Nine geochemical factors in soil and sediment samples were measured: pH, MC, TOC, TON, NH4^+^-N, NO_3_^−^-N, NO_2_^−^-N, PO_4_^3−^-P, and SiO_4_^2−^-Si (Table 1). Differences were observed in geochemical properties between submerged and unsubmerged soils. A general concentration decrease in most geochemical factors was noted from the bank slope toward submerged sediments, except for NH_4_^+^-N and SiO_4_^2−^-Si, which increased. pH and were the only two factors that significantly differed among the sites (Kruskal Wallis test, *P*<0.05). Some variations were observed within each study site, particularly in TOC, TON, NH_4_^+^-N, and PO_4_^3−^-P; TON and PO_4_^3−^-P were not detected at the Hil16, Dow16, or Sed16 site. A third pattern was the opposite changes between increasing NH_4_^+^-N and decreasing NO_3_^−^-N and NO_2_^−^-N, which suggested a role for denitrification activity operating differently between bank soils and sediments.

### 16S rRNA gene sequencing

A total of 871,521 paired-end raw reads for all samples were obtained, and 80% passed quality control and were incorporated into effective sequences. On average, each sample had 11,566 unique reads and 2,609 OTUs (Supporting Information Fig. S2). Each sample had a high sequencing depth (approximately 12,401 total reads). A rarefaction curve for each sample showed that OTUs were able to represent the overall community studied at each site (Supporting Information Fig. S3). All samples collectively represented species diversity in the study area, as shown by the species accumulation box plot (Supporting Information Fig. S4). The three replicate samples shared approximately 85% of the OTUs on average (Supporting Information Fig. S5). All four sites shared 1,716 OTUs; Hil16, Up16, and Dow16 each had approximately 3% (2.8–3.3%) unique OTUs, while Sed16 had 12% (Supporting Information Fig. S6).

### Sediment microbiome structure

The diversity of Sed16 differed from those of Dow16 and Hil16 in terms of the Chao1, ACE (Abundance-based Coverage Estimator), or Shannon Diversity Index (Wilcoxon test, *P*<0.05; Supporting Information Table S1), while these metrics did not differ between Hil16, Up16, and Dow16 samples (Wilcoxon test, *P*>0.05; Supporting Information Table S1).

**Fig. 2:**
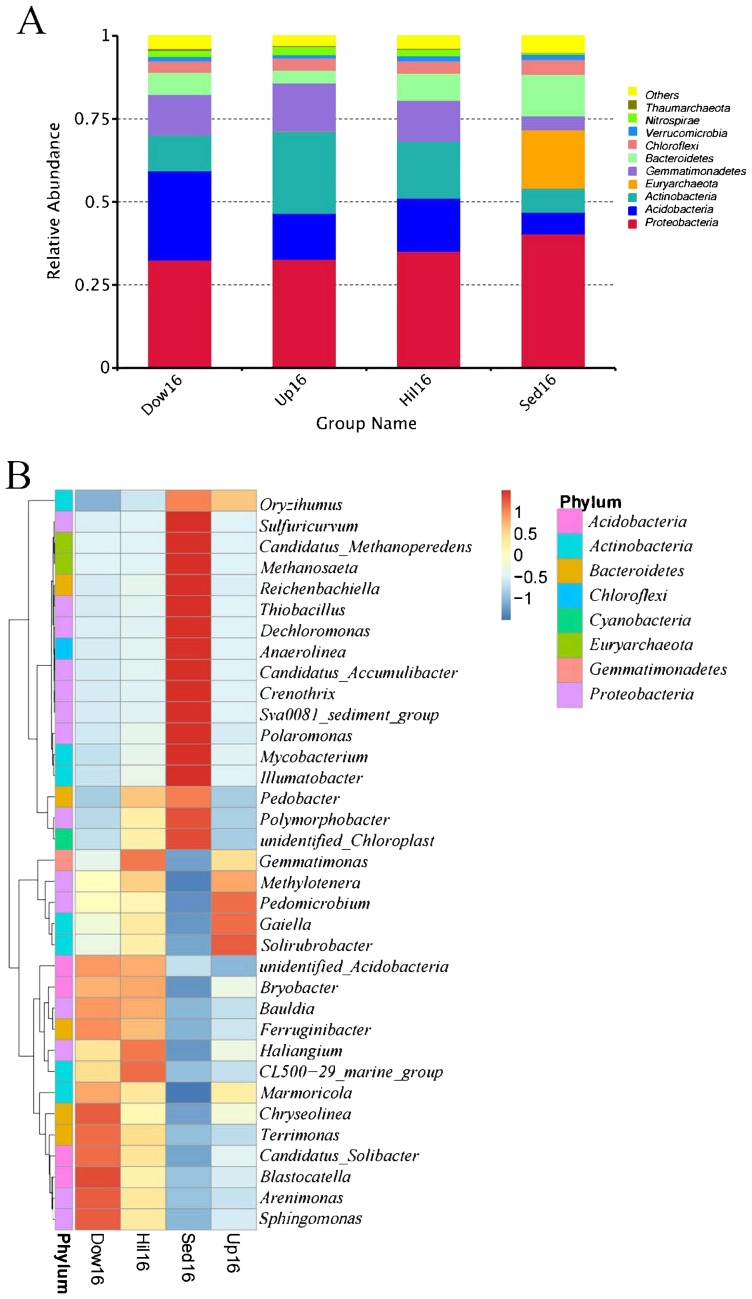
A comparison of the relative abundance of taxa between sediments and bank soils. The abundance of the top ten identified phyla (A) and a heatmap of the top 35 abundant genera (B) are indicated across the four study sites. The color scale in (B) represents the *z* score of each genus.

We also compared community compositions between sediments and soils on the lake bank. At the phylum level, Sed16 and three non-sediment sites shared most of the phyla, including some abundant phyla: *Proteobacteria*, *Actinobacteria*, *Bacteroidetes*, *Acidobacteria*, *Gemmatimonadetes*, *Nitrospirae*, and *Chloroflexi*. Sed16 had a unique phylum, *Euryarchaeota*, and non-sediment sites shared the phylum *Thaumarchaeota*, which was only present in soils (Fig. 2A). We then compared the 35 top genera that made up at least 50% of the total relative abundance and were also present in all samples between sediment and bank soil samples. Genus abundance was generally opposite; approximately half of the genera (29) were more abundant in sediment than in soil samples, while the remainder were more abundant in soil samples (Fig. 2B). The most abundant sediment-specific genera included *Sulfuricurvum*, *Methanosaeta*, *Reichenbachiella*, *Thiobacillus*, *Dechloromonas*, *Anaerolinea*, *Crenothrix*, *Polaromonas*, *Mycobacterium*, and *Ilumatobacter*. Among these genera, *Methanosaeta* only contained anaerobic species capable of utilizing acetate as their sole source of energy, and *Polaromonas* is known to live at low temperatures. Among soil-specific genera, *Pedomicrobium* and *Methylotenera* were manganese-oxidizing and methylotrophic, respectively.

Besides direct comparisons between sediments and bank soils at the phylum and genus levels, a taxonomic tree of taxa based on abundance was also provided. Among the top 70 abundant taxa, only ten were shared among the four sites and 50% were unique to each site (Fig. S7–S10).

**Fig. 3:**
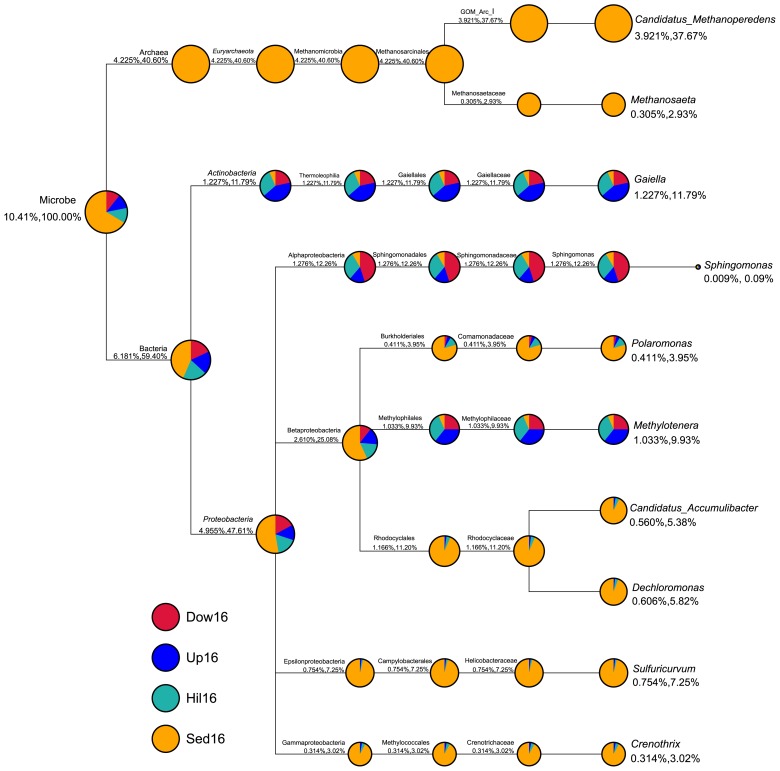
Taxonomic tree of ten most abundant genera across four study sites. The tree shows the taxonomic relationship of the ten genera; the sizes of the circles correspond to the relative abundance of this genus among all genera of the four study sites. The colors of the pie charts represent different study sites and the sizes of the pie charts represent the relative proportion of each site in the taxon specified on top of it. The two percentages under each circle represent the average relative abundance of all samples in the corresponding taxon: the first percentage shows the average abundance against all genera and the second shows the average abundance in all genera selected in this plot.

We also compared differences between the compositions of sediment and soil microbiomes with a focus on the ten most abundant genera across all four sites (Fig. 3). These ten genera accounted for 10.41% of all genus sequence abundance. Seven out of the ten genera were exclusively or primarily in the sediments (Sed16); specifically, *Candidatus*, *Methanoperedens*, and *Methanosaeta* were only present at the Sed16 site, while *Polaromonas*, *Candidatus Accumulibacter*, *Dechloromonas*, *Sulfuricurvum*, and *Crenothrix* were predominantly present in Sed16. The remaining three genera, *Methylotenera*, *Gaiella*, and *Sphingomonas*, were more abundant in soil than in sediment samples.

### Microbiome dissimilarities between sediment and soil samples

**Fig. 4:**
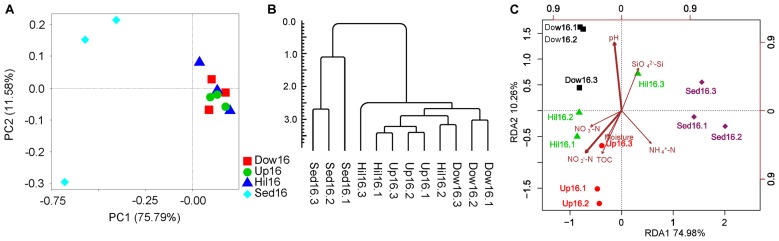
Beta diversity of microbiomes and analyses. The analyses include (A) a Principal Coordinate Analysis, (B) UPGMA tree, and (C) RDA analysis. (A) and (B) are based on weighted UniFrac distances, and (C) is based on relative OTU abundance. The scale bar in (B) indicates the tree distance; the bold arrows in (C) indicate significant explanatory factors.

Dissimilarities between samples using PCoA were examined based on weighted or unweighted UniFrac distances. As expected, sediment samples were clearly separate from soil samples, which clustered together. Along the second principal component, the three Sed16 samples were also separate from one another (Fig. 4A). This dissimilarity in samples was close to an Unweighted Pair-group Method with Arithmetic Mean (UPGMA) tree based on the weighted UniFrac distance (Fig. 4B). Differences based on UniFrac distances were confirmed by a perMANOVA test (R^2^=0.74, *P*<0.0014).

To clarify whether microbiomes were associated with geochemical parameters, RDA was performed to fit the ordination of the OTUs to seven parameters (Table 2). Sediment and soil samples were separated on the first principal component, which included nitrogen, organic carbon, and moisture. The soil samples themselves were separated on the second principal component, which mainly included pH, Si, and other factors, such as MC and TOC in the first principal component (Fig. 4C).

### Functional differences between soil and sediment microbiomes

**Fig. 5:**
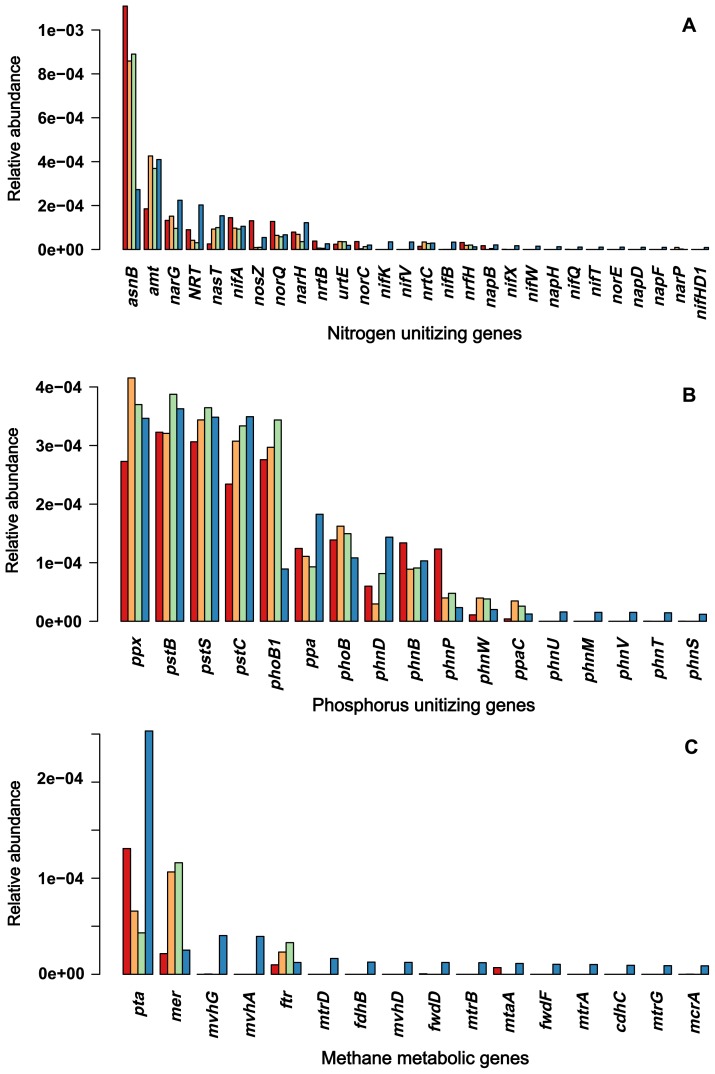
Abundance of genes involved in nitrogen and phosphorus utilization. The absolute abundance of genes involved in the metabolism of nitrate/nitrite (A), phosphate (B), and methane (C) are grouped based on function, as distinguished by the horizontal color bar on top of the gene names. The functions of the genes are explained in the text. The colored bars from left to right are: Hil16, Up16, Dow16, and Sed16.

Based on the close correlation between nitrite (NO_2_^−^-N), nitrate (NO_3_^−^-N), and microbiomes, we sequenced metagenomes to identify the genes involved in the utilization of nitrate and nitrite. The results obtained showed that each sample had approximately 2,609 OTUs on average and shared 1,716 OTUs. In the entire process of nitrate/nitrite utilization, reduction to ammonia, and assimilation to amino acids, we found genes encoding transporters for nitrate/nitrite/ammonia (NRT, *ntrABC*, and *amt*), reductase enzymes (*nap* genes, *nasA*, *nasB*, *nrfA*, *nrfH*, and *narI*), regulators (*narL*, *narX*, and *narP*), and ligases/transferases (*asnA*, *aspA*, and *glnE*) for incorporating ammonia into amino acids. These genes also included the regulatory genes (*narL*, *narX*, and *narP*) of nitrogen utilization and nitrogen fixation enzymes (*nif genes*) (Fig. 5A). In terms of abundance, these genes were generally more abundant in sediment than in soil samples, particularly those involved in nitrate/nitrite uptake (permeases and ABC transporters), intracellular nitrate/nitrite reduction, and incorporation into glutamate and glutamine. Nitrogen fixation genes were only present in sediment samples. In contrast, the genes involved in phosphate utilization were not significantly different between sediment and soil samples. These genes encoded transporters (*pstSCAB*, *phoU*, and *phnCDE*), regulators (*phoBR* and *phoH/L*), and response regulators (*cusR* and *ompR*) (Fig. 5B). Unlike nitrogen-utilizing genes, phosphate-utilizing genes showed similar levels of abundance across the four sites along the slope continuum from Hil16 to Sed16. The between-site difference between nitrogen and phosphorus utilization was significant, as confirmed by the Wilcoxon rank-sum test on coefficients of variation (*W*=514, *P*=1.1e-05).

Another difference was the presence of archaea in sediment samples. We investigated whether methane metabolism differed between sediment and soil samples. We examined the abundance of genes involved in methane metabolism, including genes converting CO_2_ to methane, methanol to methane, and acetate to methane, based on KEGG modules. We found more methane-metabolic genes in the sediments than at other sites, while no manganese-oxidizing genes were detected in any of the samples. These genes were shown in Fig. 5C. The between-site difference between methane metabolism and phosphorus utilization was significant, as confirmed by the Wilcoxon rank-sum test on coefficients of variation (*W*=497, *P*=2.3e-04).

### Microbiome modules associated with geochemical parameters

**Fig. 6:**
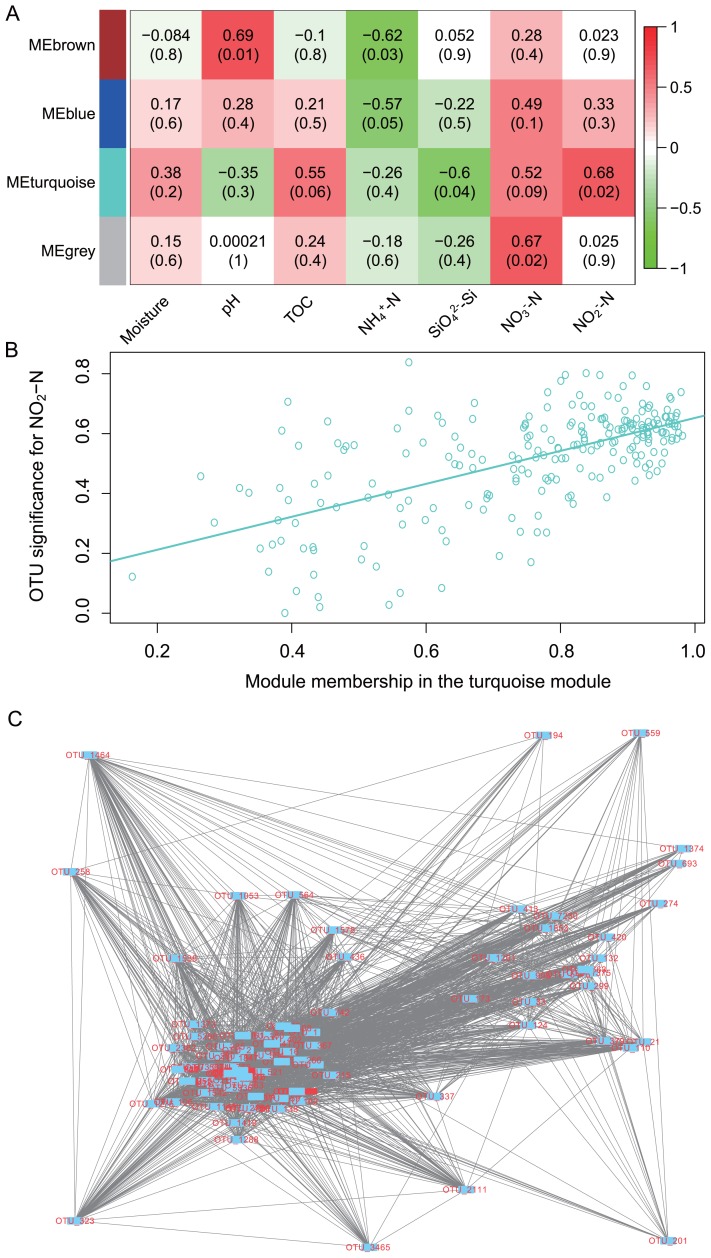
Identified modules that are closely associated with ecological factors. (A): Modules of OTUs associated with seven examined environmental factors. “ME” in the module names in the row of labels stands for module eigengene; colors distinguish modules. Numbers in the heatmap cells indicate Pearson’s correlation coefficients (top) and values in the parentheses are *P* values for the significance of the correlation. (B): The correlation between the OTU significance of the turquoise module and module membership: Spearman’s coefficient 0.62, *P*=1.7 e-15. The X axis is the module membership of OTUs in the module and the Y axis is the correlation of each module OTU with NO_2_^−^-N. (C): The topology of the turquoise module associated with NO_2_^−^-N.

Based on 2 explanatory factors (pH and NO_2_^−^-N), the modules (subnetworks of OTUs) associated with each of the examined parameters were extracted. Five microbial modules were obtained, which each correlating with NO_2_^−^-N (1; turquoise module), pH (1), NH_4_^+^-N (1), SiO_4_^2−^-Si (1), and NO_3_^−^-N (1) (Fig. 6A). NO_3_^−^-N and NO_2_^−^-N each had a significant module and another module of high correlation efficiency, but low significance. The correlations between modules and NH_4_^+^-N and SiO_4_^2−^-Si were all negative. The turquoise module closely associated with NO_2_^−^-N (Pearson’s correlation coefficient 0.62, *P*=1.72e-25) had 227 nodes (OTUs) (Fig. 6B). Twenty-five hub nodes (nodes with the most connections with neighbor nodes) were found within this module, and connected to 84 immediate neighbor nodes (nodes directly connected to the hub nodes). The module subnetwork diameter (largest shortest path length between any two nodes) and shortest path length (between any two nodes) were 2 and 1.75, respectively, which showed the tightness of connections (Fig. 6C). Fully connected nodes in the module with a clustering coefficient of 1 included g-*Ramlibacter*, f-Microbacteriaceae, g-*Rhodococcus*, g-*Caulobacter*, g-*Haliscomenobacter*, f-Rhodobiaceae, g-*Polaromonas*, g-*Illumatobacter*, g-*Rhizomicrobium*, g-*Actinocorallia*, g-*Streptomyces*, and s-*Bradyrhizobium elkanii*; all these nodes were coded in red in Fig. 6C.

## Discussion

Global warming has far-reaching impacts on the planet, including arctic ecosystems (2, 5). Increasing ice thaw has led to large-scale landscape changes, particularly post land sub-mergence. We herein examined microbiome structures and functions in sediments as indicators of climate change (18) at an arctic thaw lake. We observed changes in the bank slope extending to sediments in geochemistry, community compositions, and the gene repertoire between sediments and bank soils; these microbiomes appeared to be shaped by edaphic factors, such as NO_2_^−^-N and pH, and formed modules associated with these factors. Additionally, the relevance of NO_2_^−^-N was supported by genes involved in nitrogen utilization.

Tundra lakes have started to shrink and even disappear in high arctic regions (https://cran.r-project.org/), and thaw lakes and ponds form or expand in low arctic and subarctic regions (9). We herein reported differences in sediment microbiomes from their neighboring bank soil microbiomes. Regarding community compositions, changes were noted in diversity, dominant taxa, unique biomarker taxa, and sample dissimilarities. Although we observed some level of variation within soil samples, the main difference observed was between sediments and bank soils. This community change may also be used as an indicator of the short- and long-term effects of land submerging due to global warming. Some compositional changes have been noted in existing work on a thaw lake in the Canadian High Arctic (13, 14) and our previous study in this area (32). General continuity between soil sites and the sediment site suggests that water coverage was not only manifest in the sediments, but also in these neighboring unsubmerged microbiomes.

Besides a detailed characterization of the differences between soil and sediment microbial communities, another major result is the correlation between geochemistry and microbial communities. The continuity between soils and sediments in geochemistry and microbial communities in the present study and others (10) suggests that microbiomes respond very slowly to submergence. Furthermore, local edaphic factors, rather than global factors, have been identified as the main direct shaping force of arctic microbiomes (40); local edaphic factors include components such as pH, moisture, carbon, nitrogen, phosphorus, and trace elements (17, 34, 45, 46). pH and NO_2_^−^-N were the two major structuring factors identified by the network analysis. Most importantly, we provided a link between geochemistry and the microbial community by establishing the presence of a relationship between subnetworks and geochemical factors and by identifying the set of genes involved in denitrification. On a functional level, the high abundance of denitrification genes in sediment samples appeared to accelerate nitrite reduction, leading to the high ammonium content and low nitrite content observed. Although we did not identify the genes for the pH response, which may be very complex in microbiomes (37), we obtained some evidence to show that the genes involved in nitrogen utilization also display abundance, similar to the different abundance of taxa between sediments and soils. Further support for this functional link is that genes for phosphorus utilization do not show this sediment-soil specific pattern of abundance. The importance of nutrients, including nitrite, has already been demonstrated for microbial communities in ice melt ponds (7). We also found modules negatively associated with NH_4_^+^-N, which are the OTUs/microbes affected. The opposite changes from sediments to soils in NH_4_^+^-N/nitrate suggest a potential level of denitrification, which has been verified by the abundance of denitrifying genes in metagenomes. Similar thaw studies reported rapid shifts in many microbial functional gene abundances and pathways (29). Although nitrogen metabolism was characterized in the present study, the roles of other significant factors in microbiome changes (*e.g*., SiO_4_^2−^-Si) warrant further study. The geochemical factors that correlated with modules in the network analysis conducted in the present study did not contradict the significant factors obtained in the RDA analysis because they were identified on local (module) and global (all OTUs) levels, respectively. On the other hand, the two methods complement each other.

When compared to previous findings obtained at the same sites in 2014 (3), a major change observed was a shift in the microbiome structure. Although the top three abundant phyla were still *Proteobacteria*, *Acidobacteria*, and *Actinobacteria*, *Archaea* were not detected in 2014, but became a dominant phylum in sediments in 2016 (Sed16). Many archaeal orders in the phylum *Euryarchaeota* were also found. Another change is that some of the top 35 abundant genera between the two sampling periods differed. One potential reason for the rapid shift in composition is that the dominant genus in sediments in the 2014 study, *Clostridium* (42), was no longer detected in sediments, but was present in the Up16 site. Another difference is that compared to the similarity between Dow16 and Hil16 in the present study (48) in terms of taxonomic composition, the two sites markedly differed in 2014. The mechanisms responsible for these temporal changes will also be important when studying submergence due to global warming in the Arctic.

In summary, we herein observed significant differences in geochemistry, microbial community compositions, and functions between sediments and soils. pH and NO_2_^−^-N are two significant explanatory factors of microbiomes along the soil-to-sediment gradient. The role of denitrification in structuring microbiomes is supported by the higher levels of the entire set of denitrifying genes in sediments than in bank soils. The genes for methane metabolism are also more abundant in sediments. With intensifying global warming, the thawed lake landscape in the Arctic will gradually increase, and, thus, changes in geochemistry, microbiome structures, and gene functions will become increasingly important.

## Supplementary Information



## Figures and Tables

**Table 1:** Geochemical parameters measured at four study sites. Values in parentheses are standard deviations.

Site	Type	Moisture content (%)	pH^a^	TOC (w/v%)	TON (w/v%)	NH_4_^+^-N (μg g^−1^)	NO_3_^−^-N (μg g^−1^)	NO_2_^−^-N (μg g^−1^)	PO_4_^3−^-P (μg g^−1^)	S_i_O_4_^2−^-Si^a^ (μg g^−1^)
Hil16	Soil	13.5 (0.8)	7.877^AB^ (0.216)	1.082 (0.089)	0.088 (0.006)	1.112 (0.709)	0.325 (0.28)	0.1 (0.101)	—	2.136^AB^ (0.255)
Up16	Soil	12.6 (3.4)	7.717^A^ (0.119)	0.935 (0.728)	0.117 (0.021)	1.818 (1.05)	0.391 (0.314)	0.14 (0.093)	0.025 (0.018)	1.79^A^ (0.382)
Dow16	Soil	9.7 (1.6)	8.153^B^ (0.059)	0.207 (0.046)	—	1.157 (0.233)	0.279 (0.136)	0.039 (0.017)	—	3.988^C^ (0.767)
Sed16	Sediment	9.9 (2.6)	7.883^AB^ (0.038)	0.309 (0.202)	—	2.263 (1.572)	0.02 (0.022)	0.013 (0.002)	0.012 (0.006)	3.487^C^ (0.696)

a Significantly different between the study sites in a one-way ANOVA at*P*<0.05, followed by Tukey’s HSD test. The letters A, B, and C indicate significant differences between study sites; A and B are significantly different, while AB is not significantly different from A or B.

TOC: total organic carbon; TON: total organic nitrogen. —: not detected in some or all samples.

**Table 2:** RDA results of samples from 2016. The bold lines are explanatory factors that correlated with microbiome ordination.

	RDA1	RDA2	*r*^2^	*P* (>r)
Moisture	−0.477	−0.879	0.149	0.467
**pH**	**−0.107**	**0.994**	**0.811**	**0.003**
TOC	−0.417	−0.909	0.385	0.108
NH_4_^+^-N	0.669	−0.743	0.336	0.129
S_i_O_4_^2−^-Si	0.367	0.930	0.363	0.144
NO_3_^−^-N	−0.893	−0.451	0.210	0.348
**NO_2_^−^-N**	**−0.653**	**−0.757**	**0.528**	**0.031**
