# Supplementary material for: Geochemical-Compositional-Functional Changes in Arctic Soil Microbiomes Post Land Submergence Revealed by Metagenomics
Source: Microbes Environ. 2019 Jun 7;34(2):180–90. doi: 10.1264/jsme2.ME18091 (PMC6594734; doi:10.1264/jsme2.ME18091)
Supplement: Supplementary file 1 [file 34_180_s1.pdf]

Table S1. The comparison of alpha diversity in terms of ACE, Chao1, and Shannon index four study sites. *: significant at 0.05 level; ns: not significant.

| | Pairwise comparison | Mean difference | *p* value | Significance | Lower condifence limit | Upper confidence limit |
|---|---|---|---|---|---|---|
| ACE | Dow16_Sed16 | -13.667 | 0.016 | * | -24.468 | -2.865 |
| | Dow16_Hil16 | -9.667 | 0.077 | ns | -20.468 | 1.135 |
| | Dow16_Up16 | -3.667 | 0.486 | ns | -14.468 | 7.135 |
| | Hil16_Sed16 | -4.000 | 0.448 | ns | -14.801 | 6.801 |
| | Hil16_Up16 | 6.000 | 0.259 | ns | -4.801 | 16.801 |
| | Sed16_Up16 | 10.000 | 0.068 | ns | -0.801 | 20.801 |
| | | | | | | |
| Chao1 | Dow16_Sed16 | -15.667 | 0.005 | ** | -26.128 | -5.205 |
| | Dow16_Hil16 | -10.000 | 0.060 | ns | -20.461 | 0.461 |
| | Dow16_Up16 | -4.667 | 0.362 | ns | -15.128 | 5.795 |
| | Hil16_Sed16 | -5.667 | 0.271 | ns | -16.128 | 4.795 |
| | Hil16_Up16 | 5.333 | 0.299 | ns | -5.128 | 15.795 |
| | Sed16_Up16 | 11.000 | 0.040 | * | 0.539 | 21.461 |
| | | | | | | |
| Shannon | Dow16_Sed16 | 4.000 | 0.414 | ns | -6.032 | 14.032 |
| | Dow16_Hil16 | -10.000 | 0.051 | ns | -20.032 | 0.032 |
| | Dow16_Up16 | -0.667 | 0.891 | ns | -10.699 | 9.365 |
| | Hil16_Sed16 | 14.000 | 0.009 | ** | 3.968 | 24.032 |
| | Hil16_Up16 | 9.333 | 0.066 | ns | -0.699 | 19.365 |
| | Sed16_Up16 | -4.667 | 0.342 | ns | -14.699 | 5.365 |

Fig.S1

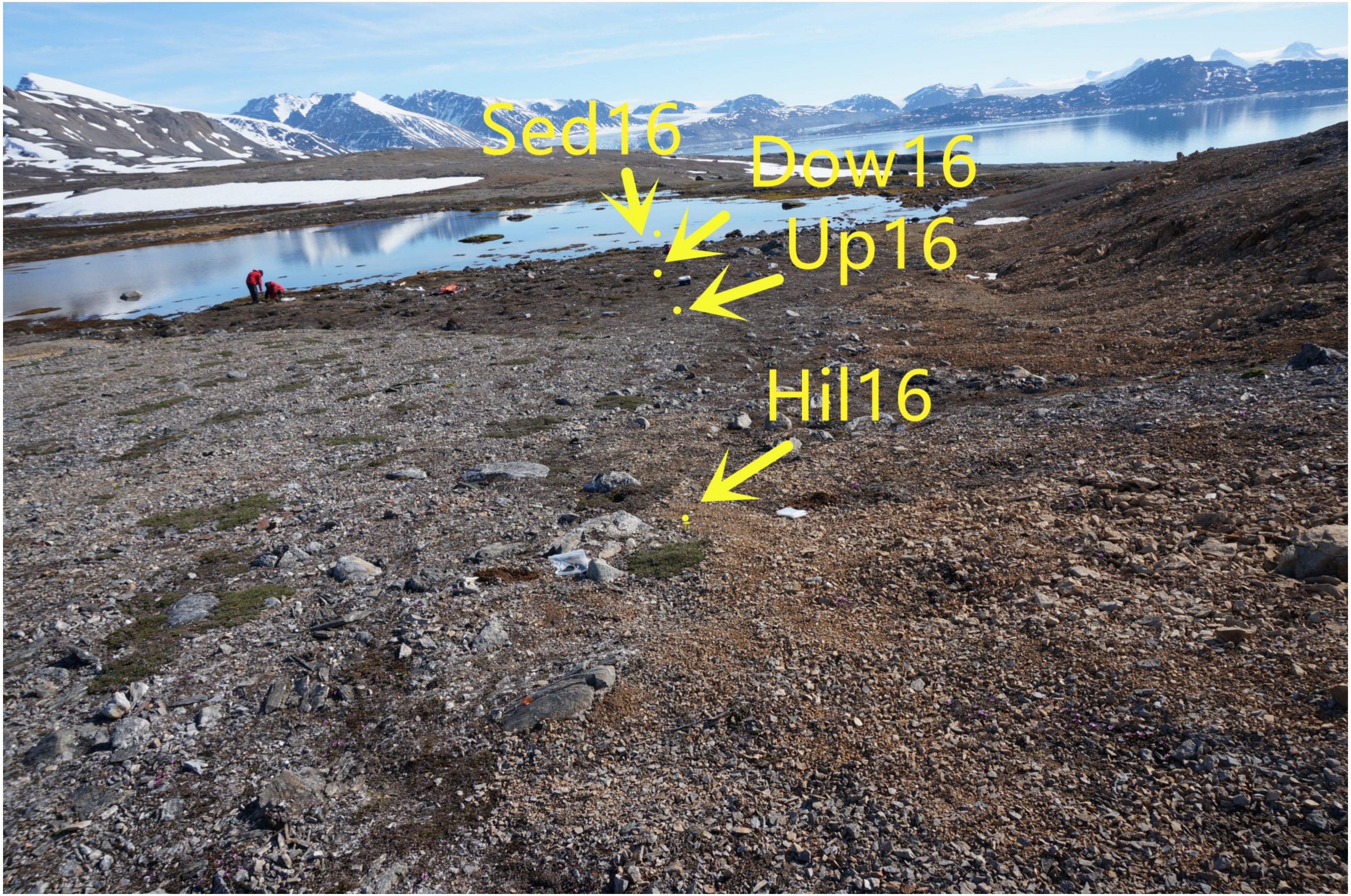

Fig.S2

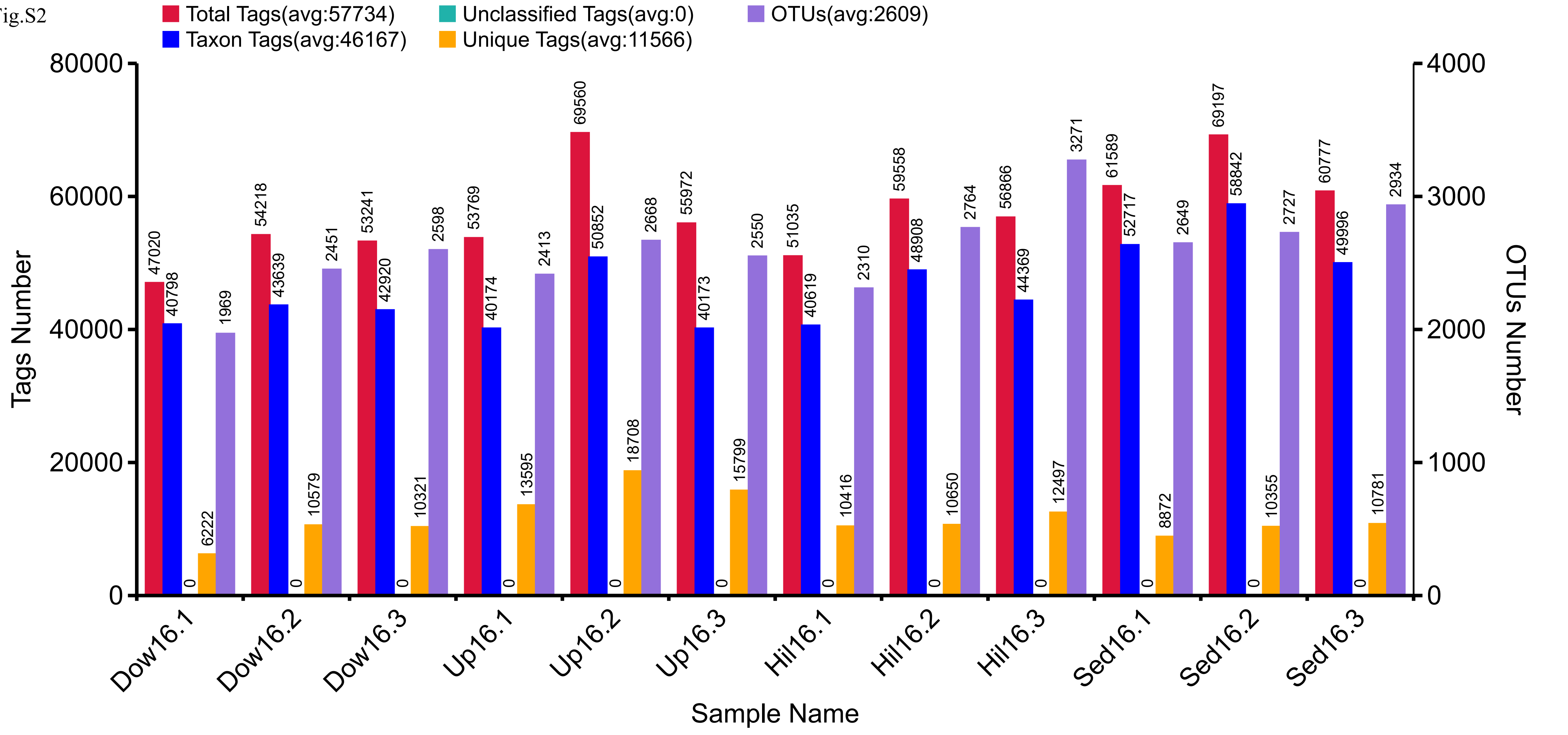
Total Tags(avg:57734)
Taxon Tags(avg:46167)
Unclassified Tags(avg:0)
Unique Tags(avg:11566)
OTUs(avg:2609)
Tags Number
OTUs Number
Sample Name
Dow16.1
Dow16.2
Dow16.3
Up16.1
Up16.2
Up16.3
Hil16.1
Hil16.2
Hil16.3
Sed16.1
Sed16.2
Sed16.3
47020
40798
0
6222
1969
54218
43639
0
10579
2451
53241
42920
0
10321
2598
53769
40174
0
13595
2413
69560
50852
0
18708
2668
55972
40173
0
15799
2550
51035
40619
0
10416
2310
59558
48908
0
10650
2764
56866
44369
0
12497
3271
61589
52717
0
8872
2649
69197
58842
0
10355
2727
60777
49996
0
10781
2934

Fig.S3

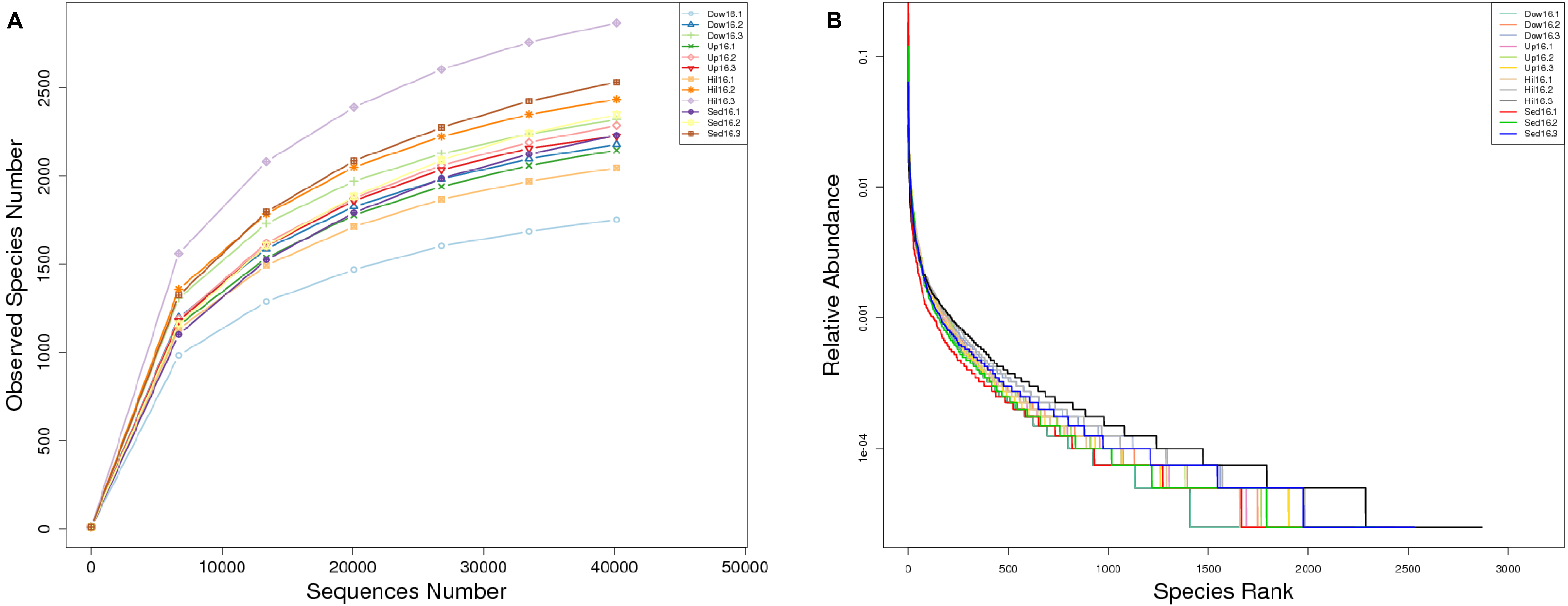
A
Observed Species Number
0
500
1000
1500
2000
2500
0
10000
20000
30000
40000
50000
Sequences Number
Dow16.1
Dow16.2
Dow16.3
Up16.1
Up16.2
Up16.3
Hil16.1
Hil16.2
Hil16.3
Sed16.1
Sed16.2
Sed16.3
B
Relative Abundance
0.1
0.01
0.001
1e-04
0
500
1000
1500
2000
2500
3000
Species Rank
Dow16.1
Dow16.2
Dow16.3
Up16.1
Up16.2
Up16.3
Hil16.1
Hil16.2
Hil16.3
Sed16.1
Sed16.2
Sed16.3

Fig.S4

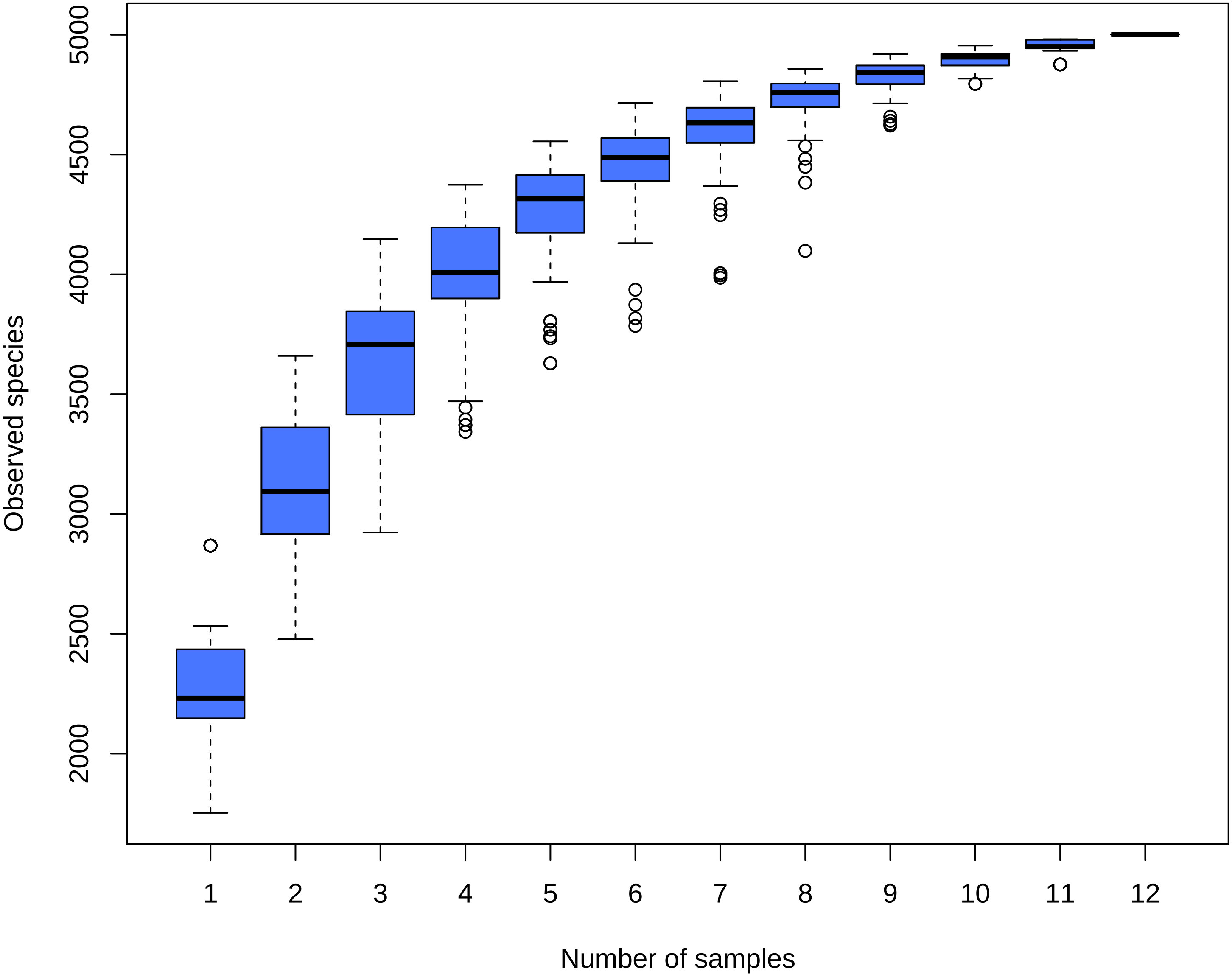

Observed species
2000
2500
3000
3500
4000
4500
5000
1
2
3
4
5
6
7
8
9
10
11
12
Number of samples

Fig.S5

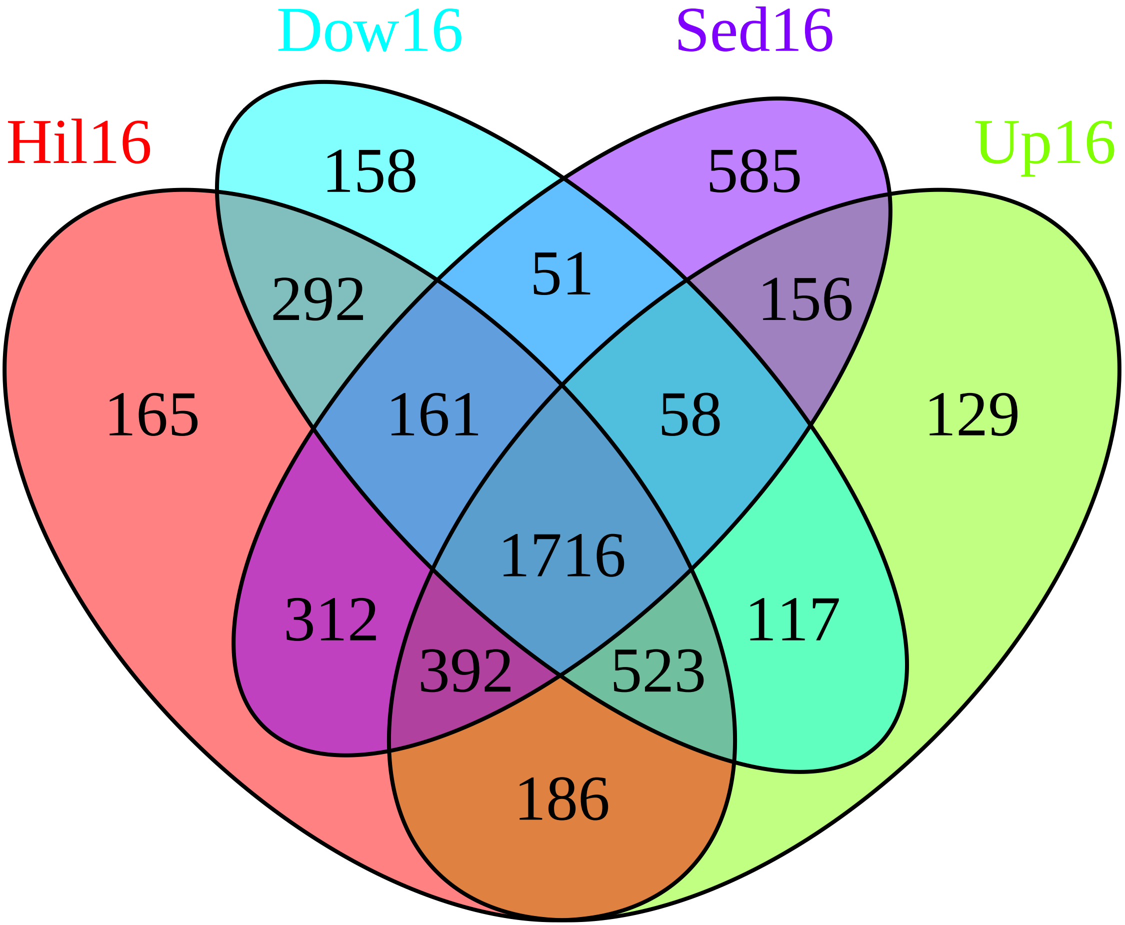
Hil16
Dow16
Sed16
Up16
158
585
51
292
156
165
161
58
129
1716
312
117
392
523
186

Fig.S6

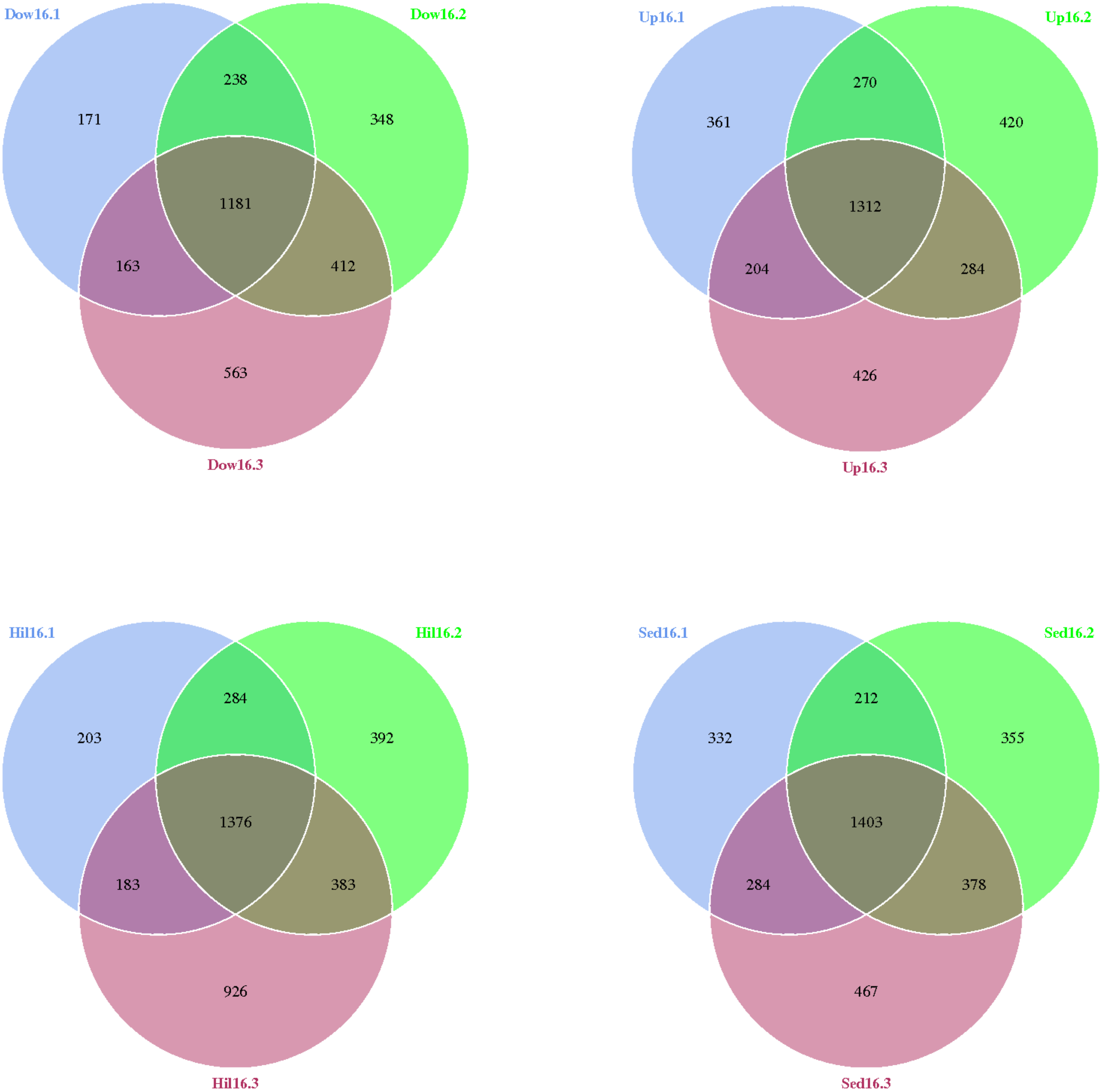

Dow16.1
Dow16.2
Dow16.3
171
238
348
1181
163
412
563
Up16.1
Up16.2
Up16.3
361
270
420
1312
204
284
426
Hil16.1
Hil16.2
Hil16.3
203
284
392
1376
183
383
926
Sed16.1
Sed16.2
Sed16.3
332
212
355
1403
284
378
467

Fig.S7

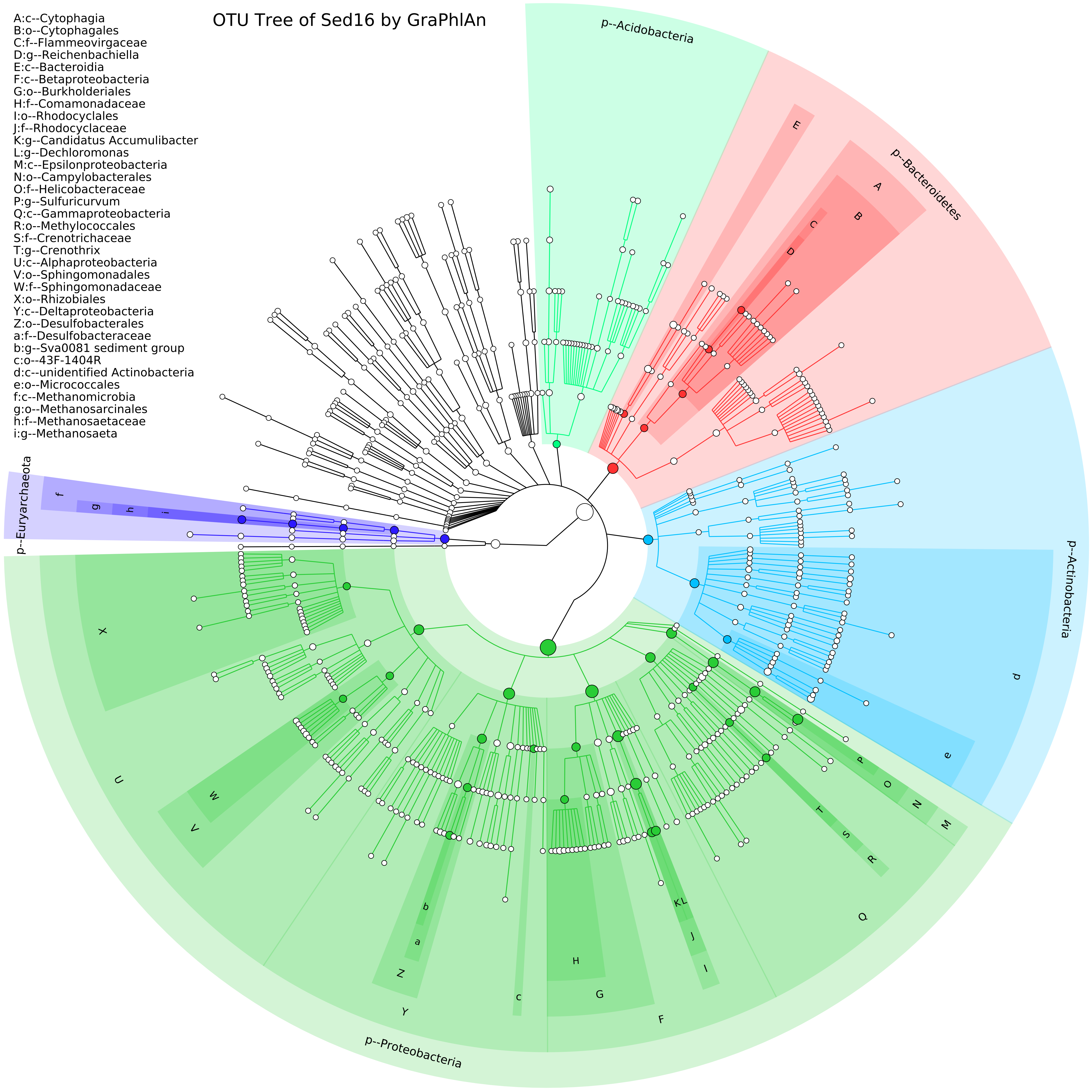
OTU Tree of Sed16 by GraPhlAn
A:c--Cytophagia
B:o--Cytophagales
C:f--Flammeovirgaceae
D:g--Reichenbachiella
E:c--Bacteroidia
F:c--Betaproteobacteria
G:o--Burkholderiales
H:f--Comamonadaceae
I:o--Rhodocyclales
J:f--Rhodocyclaceae
K:g--Candidatus Accumulibacter
L:g--Dechloromonas
M:c--Epsilonproteobacteria
N:o--Campylobacterales
O:f--Helicobacteraceae
P:g--Sulfuricurvum
Q:c--Gammaproteobacteria
R:o--Methylococcales
S:f--Crenotrichaceae
T:g--Crenothrix
U:c--Alphaproteobacteria
V:o--Sphingomonadales
W:f--Sphingomonadaceae
X:o--Rhizobiales
Y:c--Deltaproteobacteria
Z:o--Desulfobacterales
a:f--Desulfobacteraceae
b:g--Sva0081 sediment group
c:o--43F-1404R
d:c--unidentified Actinobacteria
e:o--Micrococcales
f:c--Methanomicrobia
g:o--Methanosarcinales
h:f--Methanosaetaceae
i:g--Methanosaeta
p--Acidobacteria
p--Bacteroidetes
p--Actinobacteria
p--Proteobacteria
p--Euryarchaeota

Fig.S8

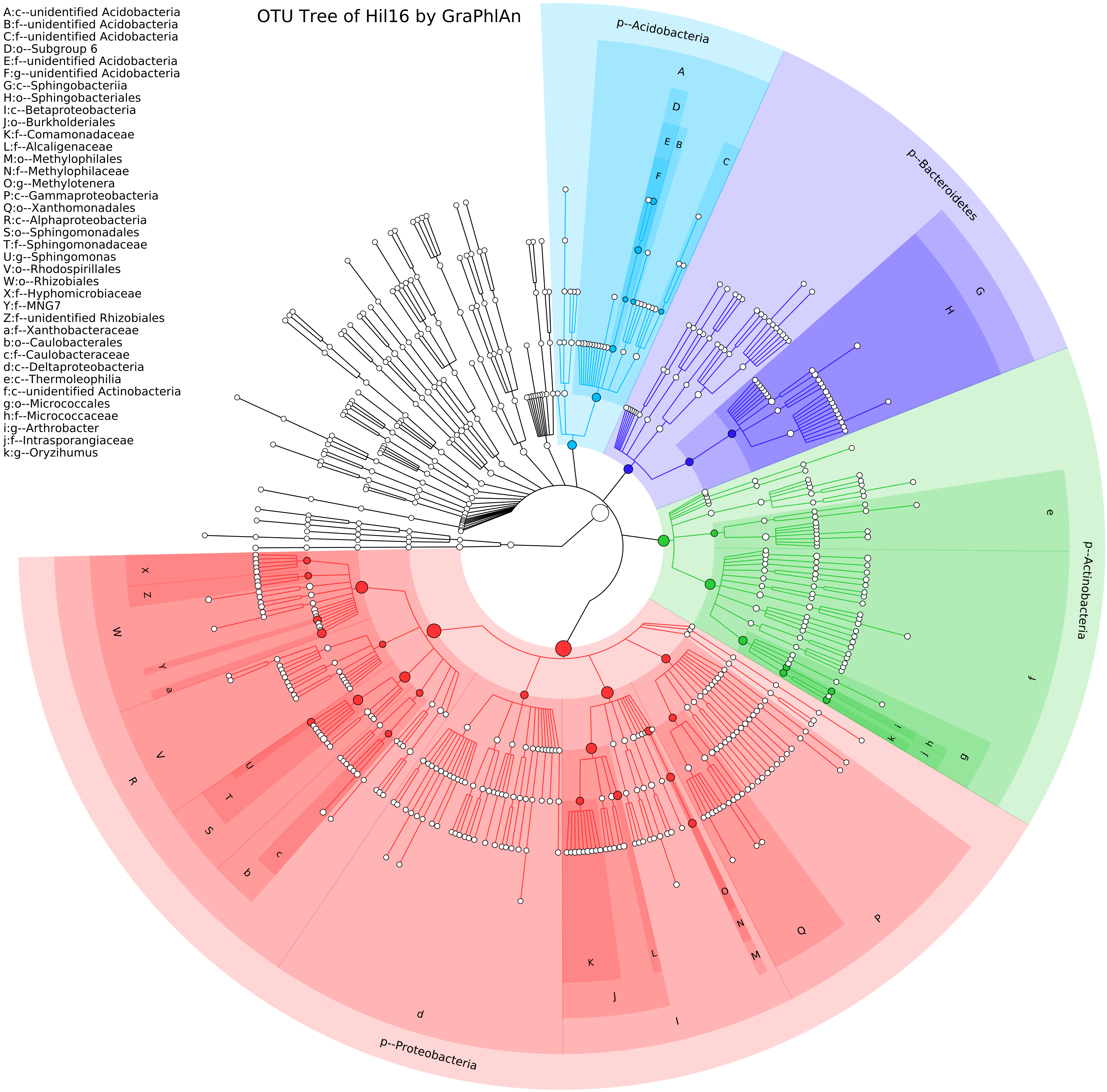

OTU Tree of Hil16 by GraPhlAn
A:c--unidentified Acidobacteria
B:f--unidentified Acidobacteria
C:f--unidentified Acidobacteria
D:o--Subgroup 6
E:f--unidentified Acidobacteria
F:g--unidentified Acidobacteria
G:c--Sphingobacteriia
H:o--Sphingobacteriales
I:c--Betaproteobacteria
J:o--Burkholderiales
K:f--Comamonadaceae
L:f--Alcaligenaceae
M:o--Methylophilales
N:f--Methylophilaceae
O:g--Methylotenera
P:c--Gammaproteobacteria
Q:o--Xanthomonadales
R:c--Alphaproteobacteria
S:o--Sphingomonadales
T:f--Sphingomonadaceae
U:g--Sphingomonas
V:o--Rhodospirillales
W:o--Rhizobiales
X:f--Hyphomicrobiaceae
Y:f--MNG7
Z:f--unidentified Rhizobiales
a:f--Xanthobacteraceae
b:o--Caulobacterales
c:f--Caulobacteraceae
d:c--Deltaproteobacteria
e:c--Thermoleophilia
f:c--unidentified Actinobacteria
g:o--Micrococcales
h:f--Micrococcaceae
i:g--Arthrobacter
j:f--Intrasporangiaceae
k:g--Oryzihumus
p--Acidobacteria
p--Bacteroidetes
p--Actinobacteria
p--Proteobacteria

Fig.S9

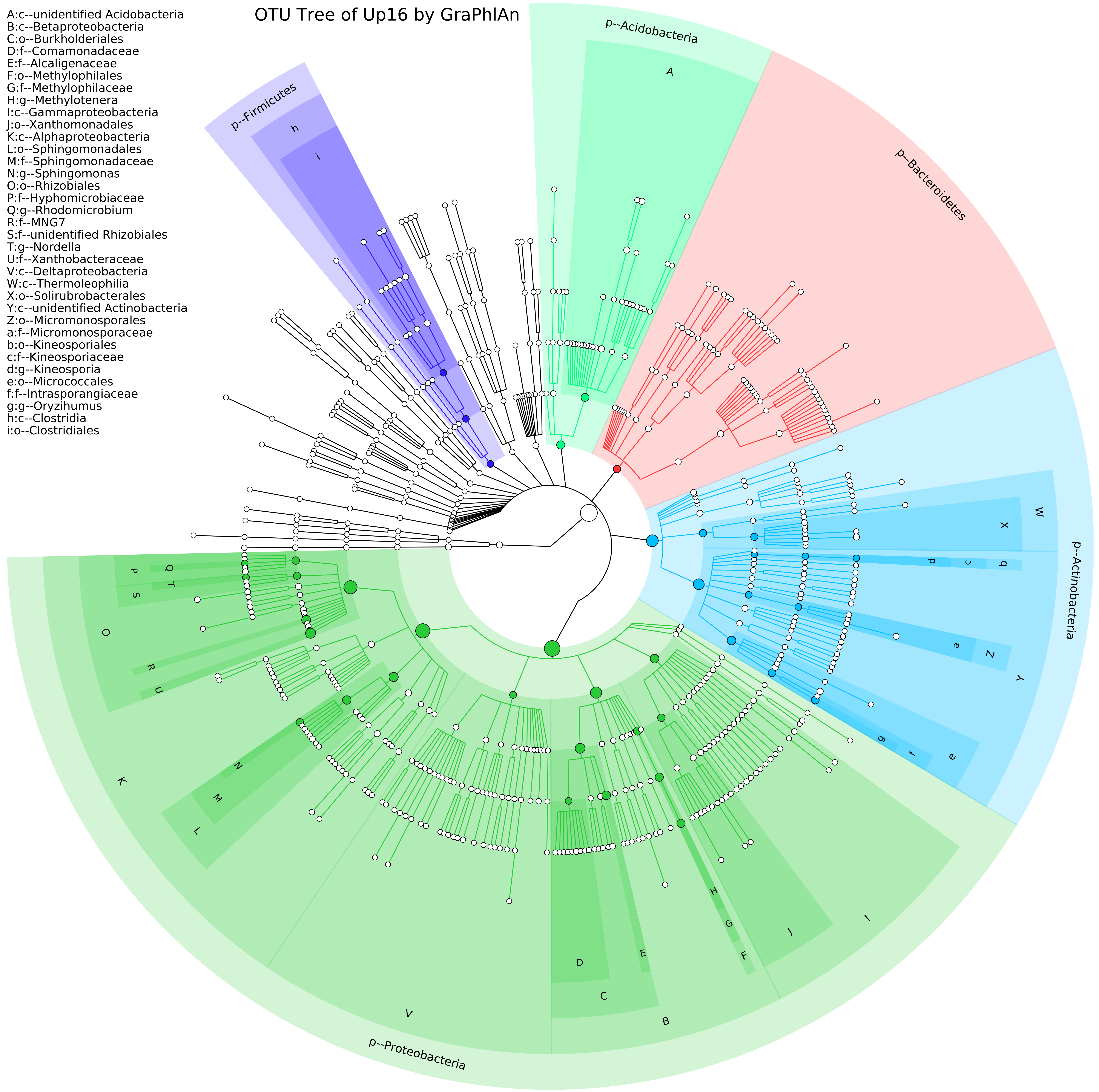

OTU Tree of Up16 by GraPhlAn
A:c--unidentified Acidobacteria
B:c--Betaproteobacteria
C:o--Burkholderiales
D:f--Comamonadaceae
E:f--Alcaligenaceae
F:o--Methylophilales
G:f--Methylophilaceae
H:g--Methylotenera
I:c--Gammaproteobacteria
J:o--Xanthomonadales
K:c--Alphaproteobacteria
L:o--Sphingomonadales
M:f--Sphingomonadaceae
N:g--Sphingomonas
O:o--Rhizobiales
P:f--Hyphomicrobiaceae
Q:g--Rhodomicrobium
R:f--MNG7
S:f--unidentified Rhizobiales
T:g--Nordella
U:f--Xanthobacteraceae
V:c--Deltaproteobacteria
W:c--Thermoleophilia
X:o--Solirubrobacterales
Y:c--unidentified Actinobacteria
Z:o--Micromonosporales
a:f--Micromonosporaceae
b:o--Kineosporiales
c:f--Kineosporiaceae
d:g--Kineosporia
e:o--Micrococcales
f:f--Intrasporangiaceae
g:g--Oryzihumus
h:c--Clostridia
i:o--Clostridiales
p--Firmicutes
p--Acidobacteria
p--Bacteroidetes
p--Actinobacteria
p--Proteobacteria

Fig.S10

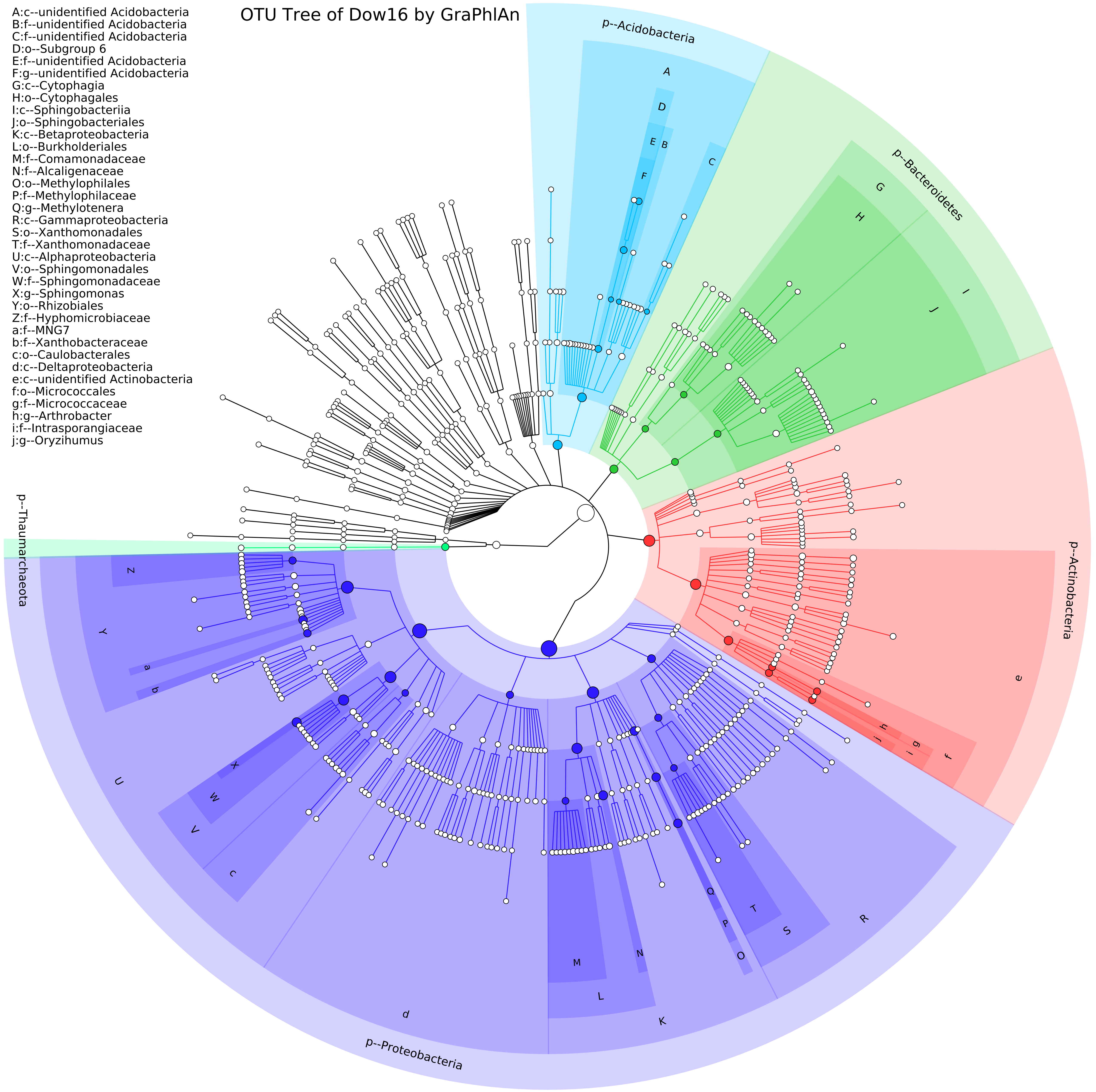
OTU Tree of Dow16 by GraPhlAn
A:c--unidentified Acidobacteria
B:f--unidentified Acidobacteria
C:f--unidentified Acidobacteria
D:o--Subgroup 6
E:f--unidentified Acidobacteria
F:g--unidentified Acidobacteria
G:c--Cytophagia
H:o--Cytophagales
I:c--Sphingobacteriia
J:o--Sphingobacteriales
K:c--Betaproteobacteria
L:o--Burkholderiales
M:f--Comamonadaceae
N:f--Alcaligenaceae
O:o--Methylophilales
P:f--Methylophilaceae
Q:g--Methylotenera
R:c--Gammaproteobacteria
S:o--Xanthomonadales
T:f--Xanthomonadaceae
U:c--Alphaproteobacteria
V:o--Sphingomonadales
W:f--Sphingomonadaceae
X:g--Sphingomonas
Y:o--Rhizobiales
Z:f--Hyphomicrobiaceae
a:f--MNG7
b:f--Xanthobacteraceae
c:o--Caulobacterales
d:c--Deltaproteobacteria
e:c--unidentified Actinobacteria
f:o--Micrococcales
g:f--Micrococcaceae
h:g--Arthrobacter
i:f--Intrasporangiaceae
j:g--Oryzihumus
p--Acidobacteria
p--Bacteroidetes
p--Actinobacteria
p--Proteobacteria
p--Thaumarchaeota
